# Therapeutic efficacy of lenvatinib in nonviral unresectable hepatocellular carcinoma

**DOI:** 10.1002/jgh3.12663

**Published:** 2021-10-22

**Authors:** Tetsu Tomonari, Yasushi Sato, Hironori Tanaka, Takeshi Mitsuhashi, Akihiro Hirao, Takahiro Tanaka, Tatsuya Taniguchi, Koichi Okamoto, Masahiro Sogabe, Hiroshi Miyamoto, Naoki Muguruma, Tetsuji Takayama

**Affiliations:** ^1^ Department of Gastroenterology and Oncology, Institute of Biomedical Sciences Tokushima University Graduate School Tokushima Japan; ^2^ Department of Community Medicine for Gastroenterology and Oncology Tokushima University Graduate School of Biomedical Sciences Tokushima Japan

**Keywords:** atezolizumab, bevacizumab, lenvatinib

## Abstract

**Aim:**

To investigate the therapeutic effect of lenvatinib (LEN) in liver disease etiology, especially nonviral hepatocellular carcinoma (HCC).

**Methods and Results:**

Sixty‐seven patients with unresectable advanced HCC (u‐HCC) treated with LEN and consisting of 26 hepatitis C virus (HCV), 19 hepatitis B virus (HBV), 11 alcohol, and 11 nonalcoholic steatohepatitis (NASH) cases were retrospectively recruited. Univariate and multivariate Cox proportional hazard models were used to determine predictive factors for survival. The objective response rate in the nonviral (alcohol and NASH) group was higher than that in the viral group (59.1% [13/22] vs. 46.7% [21/45]). Progression‐free survival was significantly longer in the nonviral group than in the viral group (13.7 vs. 6.6 months; hazard ratio [HR] 0.324; 95% confidence interval [CI] 0.174–0.602; *P* < 0.01). Similarly, median overall survival (OS) was significantly longer in the nonviral group than in the viral group (not evaluable vs. 15.9 months; HR = 0.277; 95% CI = 0.116–0.662; *P* < 0.01). Multivariate analysis revealed that portal vein invasion (HR = 5.327, *P* = 0.0025), treatment line (HR = 0.455, *P* = 0.023), and etiology (HR = 0.180, *P* = 0.00055) were significant independent factors associated with OS in u‐HCC patients treated with LEN.

**Conclusion:**

Our results suggest that LEN is more effective against nonviral u‐HCC than against viral u‐HCC.

## Graphical abstract

Kaplan–Meier analysis of progression‐free survival among patients with unresectable advanced hepatocellular carcinoma treated with lenvatinib according to etiology. The PFS in the non‐viral group was significantly longer than that in the viral group. Our results suggest that LEN is more effective against non‐viral u‐HCC than against viral u‐HCC.

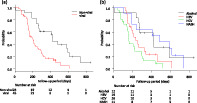

## Introduction

Hepatocellular carcinoma (HCC) is the most common primary liver cancer and the third most common cause of cancer‐related death worldwide.^1^, ^2^ Recently, the development of pharmacotherapy for unresectable advanced HCC (u‐HCC) has been remarkable, and the promising efficacy of molecular targeted therapy and combination therapy of immune checkpoint inhibitors (ICIs) and molecular targeted therapy has been reported. The Phase III SHARP trial showed that the median overall survival (OS) and disease control rates (DCR) were 10.7 months and 43%, respectively, in the group treated with sorafenib (SOR) as a primary treatment for u‐HCC.^3^ In the 10 years since then, no drug has shown an adequate survival benefit as the first‐line therapy for u‐HCC. However, the Phase III REFLECT trial showed that lenvatinib (LEN) was noninferior to SOR as the first‐line treatment for u‐HCC.^4^ LEN's effect on OS was comparable to that of SOR, which was statistically confirmed by noninferiority (median, 13.6 vs. 12.3 months; hazard ratio [HR] = 0.92, 95% confidence interval [CI] = 0.79–1.06).

More recently, the Phase III IMbrave150 trial reported that the combination of atezolizumab and bevacizumab was superior to SOR as the first‐line therapy for u‐HCC (median, not evaluable [NE] vs. 13.2 months; HR = 0.58, 95% CI = 0.42–0.79).^5^ Based on these results, the combination of atezolizumab and bevacizumab is now accepted as the first‐line treatment for u‐HCC.^6^

However, the IMbrave150 trial did not show a sufficient survival benefit, with an HR of 0.91 in patients with nonviral etiology.^5^ The efficacy of immunotherapy may be affected by the diverse hepatocyte environments that regulate hepatocyte induction and immune responses.^7^ Furthermore, ICI therapy is insufficiently effective against HCC with nonalcoholic steatohepatitis (NASH).^8^ However, the number of people who develop HCC due to nonviral etiology, such as chronic alcohol consumption and cirrhosis caused by NASH, has been increasing recently.^9^, ^10^ Therefore, optimal treatment selection for nonviral etiology is urgently required.

Although LEN was noninferior to SOR in terms of survival benefit, it is a highly effective antitumor agent.^4^, ^11^ In clinical practice, LEN is effective as the first‐line treatment and in later treatment stages. Therefore, LEN is widely used in the treatment of u‐HCC.^12^ Hepatic reserve function and relative dose intensity are predictors of therapeutic efficacy of LEN; however, there have been no reports on the efficacy of LEN focused on various etiologies of liver disease.^13^, ^14^

Therefore, we aimed to reveal the efficacy of LEN by etiology, such as its effectiveness against NASH‐, alcohol‐, and viral hepatitis‐associated HCC.

## Materials and methods

### Patient selection and HCC diagnosis

This retrospective, observational study evaluated the efficacy and safety of LEN (Lenvima, Eisai Co., Ltd., Tokyo, Japan) monotherapy in patients with u‐HCC at Tokushima University Hospital between March 2018 and January 2021. This study was approved by the Ethics Committee of Tokushima University Hospital (approval number: 3489). Written informed consent was obtained from all patients. The inclusion and exclusion criteria were based on those of the REFLECT trial. Further, Vp4, tumor volume > 50%, and non‐first‐line cases were considered as eligibility criteria. Patients with Child–Pugh class B, with performance status (PS) of ≥2, and without contrast‐enhanced computed tomography (CT) or magnetic resonance imaging (MRI) due to renal impairment or allergy were excluded. Briefly, eligible patients had target lesions defined as measurable based on the modified Response Evaluation Criteria in Solid Tumors (mRECIST),^15^ an Eastern Cooperative Oncology Group PS (ECOG PS) of 0 or 1,^16^ Barcelona Clinic Liver Cancer B or C categorizations,^1^ and Child–Pugh class A. HCC diagnosis was based on guidelines established by the Liver Cancer Study Group of Japan.^17^ Accordingly, HCC diagnosis was confirmed via histology or characteristic radiologic findings such as typical arterial enhancement of the tumor followed by a washout pattern in images of the portal venous phase or equilibrium phase obtained by dynamic spiral CT or contrast‐enhanced MRI. Regarding HCC etiology, patients with positive hepatitis B virus (HBV) surface antigen were considered to have HCC caused by HBV, and those with positive hepatitis C virus (HCV) antibodies were considered to have HCC caused by HCV. Alcoholic hepatitis was diagnosed based on a history of daily alcohol intake (>20 g for women and >30 g for men).^18^ A diagnosis of NASH required the combination of three histological features, namely steatosis, ballooning/clarification of hepatocytes, and lobular inflammation, according to a definition that has progressively gained acceptance in the liver community.^19^ Steatosis was used as the criterion for entry into the algorithm weighted by hepatocellular ballooning and lobular inflammation. A case presenting with at least grade 1 of each of the three features (steatosis, ballooning, and lobular inflammation) was classified as NASH.^19^ As patients with concealed cirrhosis often have obesity and type 2 diabetes, a substantial proportion of patients may have previously unrecognized NASH, and patients with cryptogenic cirrhosis with a body mass index of >25 kg/m^2^ and type 2 diabetes were diagnosed with burn‐out NASH.^20^, ^21^, ^22^, ^23^

### LEN treatment

The initial daily oral doses of LEN administered to patients weighing ≥60, <60, and <40 kg were 12, 8, and 4 mg/day, respectively. The initial daily oral doses of LEN provided to patients weighing ≥60 and <60 kg were 12 and 8 mg/day, respectively. For HCC patients weighing <40 kg, we started with the initial LEN dose of 4 mg/day and confirmed its safety for 1 week, which was followed by dosing up to 8 mg/day since there were no reports showing the appropriate starting dose for patients weighing <40 kg. When serious adverse events (AEs) were observed, LEN administration was discontinued. Dose interruptions were in accordance with medical package inserts for administering LEN. Briefly, when grade 3 AEs or unacceptable grade 2 AEs developed, LEN was discontinued until AEs resolved and reverted to a lower grade.

### Hepatic reserve function

Hepatic reserve function was assessed according to modified albumin–bilirubin (mALBI) grading and Child–Pugh classification. The mALBI grade was calculated based on the serum albumin and total bilirubin values using the following formula: [ALBI score = (log_10_ bilirubin [μmol/L] × 0.66) + (albumin [g/L] × −0.085)]. It was defined by the following scores: ≤−2.60 = grade 1, >−2.60 to ≤−2.27 = grade 2a, >−2.27 to ≤−1.39 = grade 2b, and > −1.39 = grade 3.^24^

### Follow‐up and patient outcomes

Patients were observed for at least 12 weeks. Safety was assessed by recording any adverse drug reactions, clinical laboratory tests, physical examination, measurement of vital signs, hematological and biochemical laboratory testing, and urinalysis. Adverse drug reactions were defined according to the Common Terminology Criteria for Adverse Events version 5.0. Radiologic responses to therapy were evaluated according to mRECIST at week 8 after starting LEN and every 8 weeks thereafter. The overall response rate (ORR) was defined as the sum of complete response (CR) and partial response (PR) rates. DCR was defined as the sum of CR, PR, and stable disease (SD) rates. Progression‐free survival (PFS) was defined as the time from the first day of administering LEN until the day of radiological progression or death from any cause.

### Statistical analyses

All statistical analyses were performed using Easy R version 1.29 (Saitama Medical Center, Jichi Medical University, Saitama, Japan).^25^ Categorical variables were compared using Fisher's exact test, and continuous variables were compared using the Mann–Whitney U and Kruskal–Wallis tests. All significance tests were two‐tailed, and statistical significance was set at a *P*‐value of <0.05. Kaplan–Meier plots of medians (with 95% CI) were used to estimate the PFS and OS. Univariate and multivariate Cox proportional hazard models were used to determine predictive factors for survival. We performed multivariate analysis using covariates such as age, sex, HCC etiology, ECOG PS, mALBI grade, number of tumors, maximum tumor size, portal vein invasion, extrahepatic metastasis, and treatment line, which are known from prior research to affect treatment outcomes in patients with HCC.^26^

## Results

### Patient characteristics

Table 1 summarizes the baseline characteristics of the study population. The median observation period after starting treatment with LEN was 453 (98–1031) days. A total of 72 patients with u‐HCC who had received LEN were enrolled in this study. However, among these, five patients were excluded because they could not be evaluated by mRECIST measurement due to renal failure. Therefore, 67 patients were retrospectively analyzed. The characteristics of these 67 patients were compared by etiology. The etiology was HCV in 26 cases; HBV, 19 cases; alcohol, 11 cases; and NASH, 11 cases. These cases were divided into viral (HCV and HBV) and nonviral (alcohol and NASH) groups for comparison. There were no significant differences in baseline characteristics between the viral and nonviral cohorts (Table S1, Supporting information).

**Table 1:** Characteristics of patients with unresectable advanced hepatocellular carcinoma treated with lenvatinib

Characteristics	All (*n* = 67)	HCV (*n* = 26)	HBV (*n* = 19)	Alcohol (*n* = 11)	NASH (*n* = 11)
Age, median [quartiles], (years)	71 [66–77]	73 [66–79]	67 [60–72]	71 [69–74]	76 [72–88]
Sex (male/female), *n*	51/16	21/5	13/6	11/0	6/5
ECOG PS (0/1), *n*	60/7	23/3	17/2	11/0	9/2
Platelets, median [quartiles], (10^4^/μL)	14.5 [8.6–19.0]	11.5 [8.6–15.9]	18.1 [13.8–20.9]	12.5 [8.8–17.6]	20.4 [14.7–24.7]
M2BpGi [quartiles] (C.O.I)	1.44 [0.95–2.50]	2.12 [1.35–3.80]	0.95 [0.66–1.89]	1.72 [1.07–3.6]	1.12 [0.85–1.39]
Child–Pugh score (5/6/7/8), *n*	38/29/0/0	18/8/0/0	9/10/0/0	6/5/0/0	5/6/0/0
mALBI grade (1/2a/2b/3), *n*	26/20/21/0	11/9/6/0	9/2/8/0	2/6/3/0	4/3/4/0
Number of intrahepatic lesions (None/1/2–7/> 7)	0/12/25/30	0/4/10/12	0/2/5/12	0/4/5/2	0/2/5/4
Maximum size of intrahepatic lesion (none/≤ 50/> 50) (mm)	0/49/18	0/19/7	0/14/5	0/9/2	0/7/4
Portal vein invasion (absent/present), *n*	54/13	22/4	16/3	9/2	8/3
Extrahepatic spread (absent/present), *n*	52/15	22/4	11/8	9/2	10/2
AFP, median [quartiles] (ng/mL)	24 [6–506]	61 [11–1878]	20 [6–1153]	30 [6–290]	15 [8–345]
BCLC stage (B/C), *n*	39/28	17/9	10/9	6/5	6/5
Treatment line (first line/second line/third line), *n*	47/10/10	16/5/5	13/3/3	10/1/0	8/1/2
Previous treatment times of TAE/TACE [quartiles]	1 [1–2]	1 [1–2]	1 [0–2]	1 [1–1]	1 [0–2]
Initial dose of Lenvatinib (12/8/4), (mg), *n*	36/30/1	11/14/1	10/9/0	8/3/0	7/4/0

AFP, alpha‐fetoprotein; ALBI, albumin–bilirubin; BCLC, Barcelona Clinic Liver Cancer; ECOG PS, Eastern Cooperative Oncology Group performance status; HBV, hepatitis B virus; HCV, hepatitis C virus; M2BPGi, mac‐2 binding protein glycosylation isomer; NBNC, non‐B non‐C; TAE/TACE, transcatheter embolization/chemoembolization.

### Treatment effect

The results of the therapeutic response according to mRECIST are presented in Table 2. There were 67 patients with measurable nodules that could be evaluated by enhanced CT/MRI 8 weeks after starting LEN treatment.

**Table 2:** Response to treatment with lenvatinib for advanced unresectable hepatocellular carcinoma according to etiology

Evaluation (mRECIST)	CR	PR	SD	PD	ORR (%)	DCR (%)
Etiology						
All (*n* = 67)	2 (3.0)	32 (47.8)	31 (46.2)	2 (3.0)	50.8	97.0
HCV (*n* = 26)	1 (3.8)	7 (26.9)	16 (65.4)	2 (7.7)	30.8	92.3
HBV (*n* = 19)	1 (5.3)	12 (63.1)	6 (31.6)	0 (0)	68.4	100
Alcohol (*n* = 11)	0 (0)	7 (63.6)	4 (36.3)	0 (0)	63.6	100
NASH (*n* = 11)	0 (0)	6 (54.5)	5 (45.5)	0 (0)	54.5	100
Viral (*n* = 45)	1 (2.2)	20 (44.4)	23 (51.1)	2 (4.4)	46.7	95.6
NBNC (*n* = 22)	0 (0)	13 (59.1)	9 (40.9)	0 (0)	59.1	100

ALBI, albumin–bilirubin; BCLC, Barcelona Clinic Liver Cancer; CR, complete response; DCR, disease control rate; HBV, hepatitis B virus, HCV, hepatitis B virus; mRECIST, modified response evaluation criteria in solid tumors; NASH, nonalcoholic steatohepatitis; ORR, overall response rate; PD, progressive disease; PR, partial response; SD, stable disease.

Among these 67 patients, 2 exhibited CR (3.0%; 2/67), 32 had PR (47.8%; 32/67), 31 had SD (46.2%; 31/67), and 2 had progressive disease (PD) (3%; 2/67). The ORR and DCR were 50.8% (34/67) and 97% (65/67), respectively.

**Figure 1:**
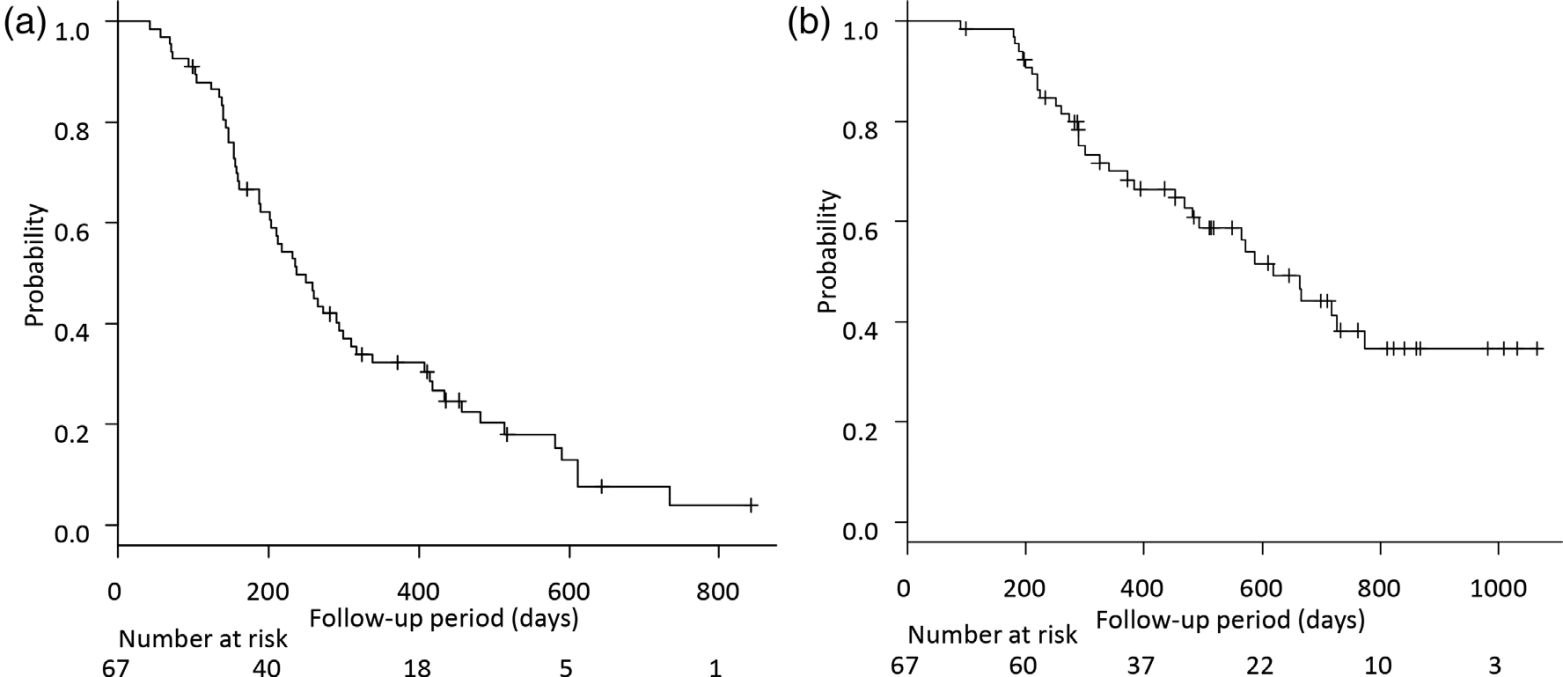
Progression‐free survival (a) and overall survival (b) among patients with unresectable advanced hepatocellular carcinoma treated with lenvatinib.

Regarding the therapeutic response in the viral and nonviral groups, the ORR in the nonviral group was higher than that in the viral group (59.1% [13/22] vs. 46.7%; 21/45], but the difference was not statistically significant (*P* = 0.31). The PFS of all patients was 7.8 (95% CI = 6.2–9.8) months (Fig. 1a). The median OS of all patients was 20.3 (95% CI = 15.4–25.4) months (Fig. 1b).

**Figure 2:**
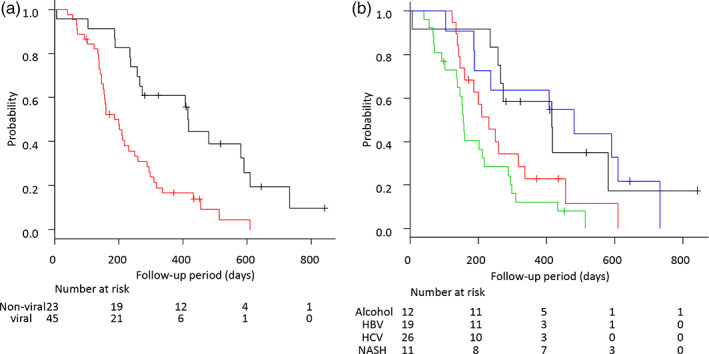
Kaplan–Meier analysis of progression‐free survival among patients with unresectable advanced hepatocellular carcinoma treated with lenvatinib according to etiology. (a) The PFS in the nonviral group was significantly longer than that in the viral group. 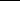, Nonviral; 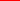, viral. (b) The PFS among alcohol, NASH, HBV, and HCV groups. HBV, hepatitis B virus; HCV, hepatitis B virus; NASH, nonalcoholic steatohepatitis; PFS, progression‐free survival. 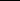, Alcohol; 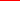, HBV; 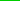, HCV; 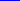, NASH.

The PFS in the nonviral group was significantly longer than that in the viral group (13.7 vs. 6.6 months; HR = 0.324; 95% CI = 0.174–0.602; *P* < 0.01; Fig. 2a), whereas PFS between the alcohol and NASH groups (nonviral group: 13.6 vs. 15.8 months; HR = 1.12; 95% CI = 0.40–3.08; *P* = 0.83) and the HBV and HCV groups (viral group; 7.6 vs. 5.1 months; HR = 1.71; 95% CI = 0.89–3.27; *P* = 0.11) showed no significant differences (Fig. 2b).

**Figure 3:**
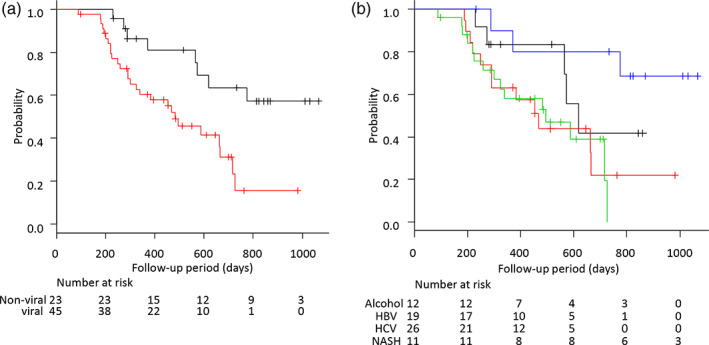
Kaplan–Meier analysis of overall survival among patients with unresectable advanced hepatocellular carcinoma treated with lenvatinib according to etiology. (a) The OS in the nonviral group was significantly longer than that in the viral group. 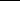, Nonviral; 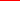, viral. (b) The OS among alcohol, NASH, HBV, and HCV groups. HBV, hepatitis B virus, HCV, hepatitis B virus; NASH, nonalcoholic steatohepatitis; OS, overall survival. 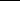, Alcohol; 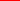, HBV; 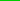, HCV; 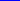, NASH.

Similarly, the median OS in the nonviral group was significantly longer than that in the viral group (NE vs. 15.9 months; HR = 0.277; 95% CI = 0.116–0.662; *P* < 0.01; Fig. 3a), whereas there were no significant differences in OS between the alcohol and NASH groups (nonviral group; 20.3 vs. NE months; HR = 0.51; 95% CI = 0.22–2.35; *P* = 0.39) and the HBV and HCV groups (viral group; 15.4 vs. 16.2 months; HR = 1.03; 95% CI = 0.48–2.21; *P* = 0.93) (Fig. 3b).

Furthermore, we analyzed both the NAFLD and non‐NAFLD groups. PFS in the NAFLD group was significantly longer than that in the non‐NAFLD group (15.8 vs. 7.6 months; HR = 2.066; 95% CI = 0.99–4.32; *P* < 0.05; Fig. S1a, Supporting information). The median OS in the nonviral group tended to be longer than that in the viral group (NE vs. 18.8 months; HR = 4.05; 95% CI = 1.20–13.6; *P* < 0.01; Fig. S1b).

In addition, we analyzed both the viral and nonviral groups in the first line of 47 patients. PFS in the nonviral group was significantly longer than that in the viral group (15.8 vs. 6.7 months; HR = 0.350; 95% CI = 0.170–0.714; *P* < 0.01; Fig. S2a, Supporting information). The median OS in the nonviral group tended to be longer than that in the viral group (NE vs. 21.9 months; HR = 0.422; 95% CI = 0.147–1.219; *P* = 0.09; Fig. S3a, Supporting information).

### Multivariate analysis of clinical factors affecting the outcome of LEN treatment

The univariate analysis performed to determine baseline prognostic factors associated with better PFS revealed that mALBI grade, portal vein invasion, treatment line, and etiology (viral/nonviral) were prognostic factors for u‐HCC patients treated with LEN (*P* = 0.006, *P* = 0.0043, *P* = 0.0002, and *P* = 0.03, respectively; Table 3). Multivariate analysis revealed that mALBI grade 1 or 2a (HR = 0.498, *P* = 0.046), portal vein invasion (HR: 3.267, *P* = 0.0057), and etiology (nonviral; HR: 0.246, *P* = 0.000021) were significant independent factors associated with PFS in u‐HCC patients treated with LEN (Table 3).

**Table 3:** Univariate and multivariate analyses of the factors influencing progression‐free survival

				Univariate	Multivariate	
Variables	Category	No. of patients	Median PFS (days)	*P* value	Hazard ratio (95% confidence interval)	*P* value
Age, (years)	≥70 <70	40 27	252 217	0.73		
Sex	Male Female	51 16	261 211	0.77		
Etiology	Nonviral Viral	22 45	418 201	0.00020	0.246 (0.129–0.470)	0.000022
ECOG PS	1 0	7 60	211 250	0.78		
mALBI grade	1,2a 2b	46 21	290 188	0.0060	0.498 (0.250–0.991)	0.046
Number of tumors	>7 ≤7	36 31	263 211	0.61		
Maximum size of tumor (mm)	≥50 <50	18 49	188 261	0.61		
Portal vein invasion	Yes No	12 55	147 261	0.0043	3.267 (1.411–7.563)	0.0057
Extrahepatic spread	Yes No	15 52	211 259	0.39		
AFP level (ng/mL)	≥400 <400	19 48	203 260	0.60		
Treatment line	First line	19	189	0.033		
	Later line	48	290			

AFP, alpha‐fetoprotein; ECOG PS, Eastern Cooperative Oncology Group performance status; NA, not applicable; OS, overall survival.

The univariate analysis performed to determine baseline prognostic factors associated with better OS revealed that mALBI grade 1 or 2a, portal vein invasion, treatment line, and etiology (nonviral) were prognostic factors for u‐HCC patients treated with LEN (*P* = 0.013, *P* = 0.0035, *P* = 0.0055 and *P* = 0.0024, respectively; Table 4). Multivariate analysis revealed that portal vein invasion (HR = 5.327, *P* = 0.0025), treatment line (HR = 0.455, *P* = 0.023), and etiology (nonviral; HR = 0.203, *P* = 0.00065) were significant independent factors associated with OS in u‐HCC patients treated with LEN (Table 4).

**Table 4:** Univariate and multivariate analyses of the factors influencing overall survival

				Univariate	Multivariate	
Variables	Category	No. of patients	Median OS (days)	*P* value	Hazard ratio (95% confidence interval)	*P* value
Age, (years)	≥70 <70	40 27	727 494	0.082		
Sex	Male Female	51 16	618 324	0.57		
Etiology	Nonviral Viral	22 45	NA 483	0.0024	0.180 (0.068–0.477)	0.00055
ECOG PS	1 0	7 60	664 618	0.82		
mALBI grade	1,2a 2b	46 21	717 295	0.013		
Number of tumors	>7 ≤7	36 31	618 565	0.44		
Maximum size of tumor (mm)	≥50 <50	18 49	572 664	0.66		
Portal vein invasion	Yes No	12 55	273 665	0.0035	5.327 (2.176–13.040)	0.0025
Extrahepatic spread	Yes No	15 52	587 665	0.41		
AFP level (ng/mL)	≥400 <400	19 48	665 618	0.77		
Treatment line	First line	48	727	0.0055	0.455	0.023
	Later line	19	371			

AFP, alpha‐fetoprotein; ECOG PS, Eastern Cooperative Oncology Group performance status; NA, not applicable; OS, overall survival.

Next, in the first‐line treatment group analysis, the univariate analysis performed to determine baseline prognostic factors associated with better PFS revealed that portal vein invasion and etiology (viral/nonviral) were prognostic factors for u‐HCC patients treated with LEN (*P* = 0.011, *P* = 0.0027, respectively; Table S2, Supporting information). Multivariate analysis revealed that portal vein invasion (HR = 4.837, *P* = 0.00092) and etiology (nonviral; HR = 0.268, *P* = 0.00059) were significant independent factors associated with PFS in u‐HCC patients treated with LEN (Table S2).

The univariate analysis performed to determine baseline prognostic factors associated with better survival revealed that portal vein invasion and etiology (nonviral) were prognostic factors for u‐HCC patients treated with LEN (*P* = 0.04, *P* = 0.0011, respectively; Table S3, Supporting information). Multivariate analysis revealed that portal vein invasion (HR = 6.816, *P* = 0.048) and etiology (nonviral; HR = 0.344, *P* = 0.048) were significant independent factors associated with OS in u‐HCC patients treated with LEN (Table S3).

### Adverse events in the LEN cohorts

Grade 4 AEs were not observed during the observation period. The most common all‐grade drug‐related AEs were hypertension (47.8%; 32/67), fatigue (47.8%; 32/67), proteinuria (46.3%; 31/67), decreased appetite (32.8%; 22/67), and palmar‐plantar erythrodysesthesia (28.4%; 19/67). The most common grade 3 drug‐related AEs were proteinuria (19.4%, 13/67), hypertension (11.9%, 8/67), fatigue (6.0%, 4/67), decreased platelet count (3%, 2/67), and diarrhea (3%, 2/67). There were no significant differences in LEN‐related AEs between the viral and nonviral groups (Table S2).

## Discussion

In this study, LEN showed significantly better PFS and OS in the nonviral HCC group than in the viral HCC group. In multivariate analysis, first‐line, portal vein invasion and nonviral etiology were independent prognostic factors. Furthermore, the analysis of the first‐line group also showed that portal vein invasion and nonviral etiology were independent prognostic factors. These results suggest that LEN is an effective therapeutic option against u‐HCC associated with nonviral etiology. Therefore, the present findings suggest that LEN could be an alternative to atezolizumab and bevacizumab in nonviral HCC.

HCC has distinct etiologic factors. Common etiologies of HCC include HBV, HCV, NASH, and alcoholic liver disease. Due to these various etiologies, the carcinogenic process differs from tumor biological characteristics. These differences may affect the efficacy of targeted therapies.^27^, ^28^, ^29^ In a subanalysis of the SHARP study, Bruix et al. reported that SOR had an inadequate prolongation of time to progression in HBV‐positive patients.^30^ However, regarding patients receiving LEN, differences in therapeutic effects among various background liver disease etiologies have yet to be evaluated.

Recently, Tsuchiya et al. reported no significant difference in PFS and OS between viral and nonviral u‐HCC patients treated with LEN in a multicenter study.^31^ However, their report included 20% of patients with Child–Pugh class B, whereas all our cases were Child–Pugh class A patients. Given that the treatment outcome of LEN is associated with hepatic reserve function, the different results may be attributed to these.^11^, ^32^

Recent progress in pharmacotherapy for u‐HCC has been remarkable, and although several drugs have shown efficacy, atezolizumab plus bevacizumab is currently recommended as the first‐line treatment.^5^, ^6^ However, the results of three large clinical trials related to immunotherapy recently reported insufficient efficacy in the group with nonviral etiology (CheckMate‐459: HR = 0.95 [95% CI = 0.74–1.22], IMbrave150: HR = 0.91 [95% CI = 0.52–1.59], KEYNOTE‐240: HR = 0.88 [95% CI = 0.77–1.1]).^5^, ^33^, ^34^ More recently, ICI treatment has been less effective for NASH‐related HCC because CD8+ positive lymphocytes in NASH‐associated HCC have a reduced immune response to cancer antigens due to reduced antitumor surveillance.^8^ The IMbrave150 study reported that SOR is effective even in nonviral conditions in terms of OS.^5^ Moreover, a sub‐analysis of the REFLECT trial showed that LEN for patients with nonviral (alcohol) u‐HCC showed better PFS than SOR (HR = 0.27 [95% CI = 0.11–0.66], 8.8 vs. 3.9 months), which suggests that LEN is an effective therapeutic option for nonviral (alcohol) HCC.

In fact, our study showed that the PFS of LEN in alcohol‐associated u‐HCC (13.7 months) was favorably comparable to the PFS of LEN in the REFLECT trial (Fig. S1b).

Although the reasons for the high efficacy of LEN against nonviral HCC are not evident in our study, this high efficacy was observed in alcohol‐associated u‐HCC and NASH‐associated u‐HCC (Fig. 2). When considering the mechanisms of action of targeted therapies, LEN is an oral molecular‐targeted agent (MTA) that targets vascular endothelial growth factor (VEGF) receptors 1–3, fibroblast growth factor receptors (FGFRs) 1–4, platelet‐derived growth factor (PDGF) receptor α, RET, and KIT.^35^, ^36^, ^37^, ^38^, ^39^ SOR is also an oral MTA that blocks RAF kinase, VEGF receptors, and PDGF receptors KIT and FLT3. LEN is unique from SOR in that it targets FGFRs 1–4. FGF19–FGFR4 pathways have been proven to be a carcinogenic driver of HCC,^40^, ^41^ and FGF19‐driven HCC may be indicated for LEN therapy.^42^, ^43^ More importantly, FGF19 and its receptor, FGFR4, are involved in the promotion of hepatic stem cells in the carcinogenesis process from fatty liver to HCC.^44^, ^45^ Alcohol consumption shares many pathophysiological processes with other forms of cirrhosis, in particular with NASH.^10^ Moreover, FGF19 can be secreted by cells from pathological liver tissue, such as cholestatic non‐cirrhotic and cirrhotic livers and livers from individuals with alcoholic hepatitis and HCC.^46^ Therefore, LEN, which is effective for FGF19‐driven HCC, may also be effective for NASH‐ and alcohol‐associated nonviral HCC. In fact, in our cohort, both the nonviral NASH‐ and alcohol‐associated u‐HCC groups showed an equally better response rate, PFS, and OS than the viral u‐HCC group; these findings were also observed when LEN was used as the first‐line treatment (Figs S1a and S2a).

Hepatic reserve function and portal vein invasion at treatment initiation are general prognostic factors for u‐HCC treatment with MTAs,^32^, ^47^ and they were also found to be prognostic factors in our study; however, the hepatic function was observed only in PFS and not in OS analysis. This may be due to the limitation of this study described below. In this study, etiology was identified as a prognostic factor for LEN treatment, which has not received much attention to date. This may become an important indicator in the selection of the initial treatment for u‐HCC, especially in deciding whether to use ICIs or MTAs.

The main limitations of our study are its retrospective nature, small sample size, and short observation period. Although analysis of the first‐line treatment also showed that LEN resulted in better survival in the nonviral HCC group than in the viral HCC group, the number of these cases may be insufficient to show the efficacy of LEN as the first‐line treatment against nonviral u‐HCC. Therefore, a large‐scale prospective study is required to confirm the present findings and to conduct more detailed analyses, such as a prospective comparison of LEN and ICIs in patients with nonviral hepatitis‐derived u‐HCC.

## Conclusion

Our results suggest that LEN treatment showed better clinical efficacy in nonviral hepatitis‐associated u‐HCC than in viral hepatitis‐associated u‐HCC, indicating that we should consider etiology when treating u‐HCC. However, further studies are required to confirm the effect of LEN on u‐HCC based on etiology.

## Author Contributions

Tetsu Tomonari, study concept and design, acquisition of data, statistical analysis, and drafting of the manuscript. Yasushi Sato, statistical analysis and revision of the manuscript. Hironori Tanaka, acquisition of data. Takahiro Tanaka, acquisition of data. Tatsuya Taniguchi, acquisition of data. Koichi Okamoto, acquisition of data. Masahiro Sogabe, acquisition of data. Hiroshi Miyamoto, acquisition of data. Naoki Muguruma, acquisition of data. Tetsuji Takayama, revision of the manuscript. All authors had access to the data and participated in the writing of this manuscript.

## Supporting information


**Figure S1.** Kaplan–Meier analysis of progression‐free survival among patients with advanced hepatocellular carcinoma treated with lenvatinib as the first‐line treatment according to etiology. (a) The PFS in the non‐NAFLD group was significantly longer than NAFLD group. Kaplan–Meier analysis of overall survival among patients with advanced hepatocellular carcinoma treated with lenvatinib as the first‐line treatment according to etiology. (b) The OS in the nonviral group was significantly longer than that in the viral group. NAFLD, nonalcoholic fatty liver disease; OS, overall survival.

**Figure S2.** Kaplan–Meier analysis of progression‐free survival among patients with advanced hepatocellular carcinoma treated with lenvatinib as the first‐line treatment according to etiology. (a) The PFS in the nonviral group was significantly longer than that in the viral group. (b) The PFS among alcohol, NASH, HBV, and HCV groups. NASH, nonalcoholic steatohepatitis; HBV, hepatitis B virus; HCV, hepatitis C virus; PFS, progression‐free survival.

**Figure S3.** Kaplan–Meier analysis of overall survival among patients with advanced hepatocellular carcinoma treated with lenvatinib as the first‐line treatment according to etiology. (a) The OS in the nonviral group was significantly longer than that in the viral group. (b) The OS among alcohol, NASH, HBV, and HCV groups. HBV, hepatitis B virus; HCV, hepatitis C virus; OS, overall survival.

**Table S1.** Characteristics of patients with unresectable advanced hepatocellular carcinoma treated with lenvatinib.
**Table S2.** Univariate and multivariate analyses of the factors influencing progression‐free survival in first line.
**Table S3.** Univariate and multivariate analyses of the factors influencing overall survival in first line.
**Table S4.** Adverse events associated with lenvatinib treatment.
