# Supplementary material for: Therapeutic efficacy of lenvatinib in nonviral unresectable hepatocellular carcinoma
Source: JGH Open. 2021 Oct 22;5(11):1275–83. doi: 10.1002/jgh3.12663 (PMC8593789; doi:10.1002/jgh3.12663)

Supplementary.1

a)

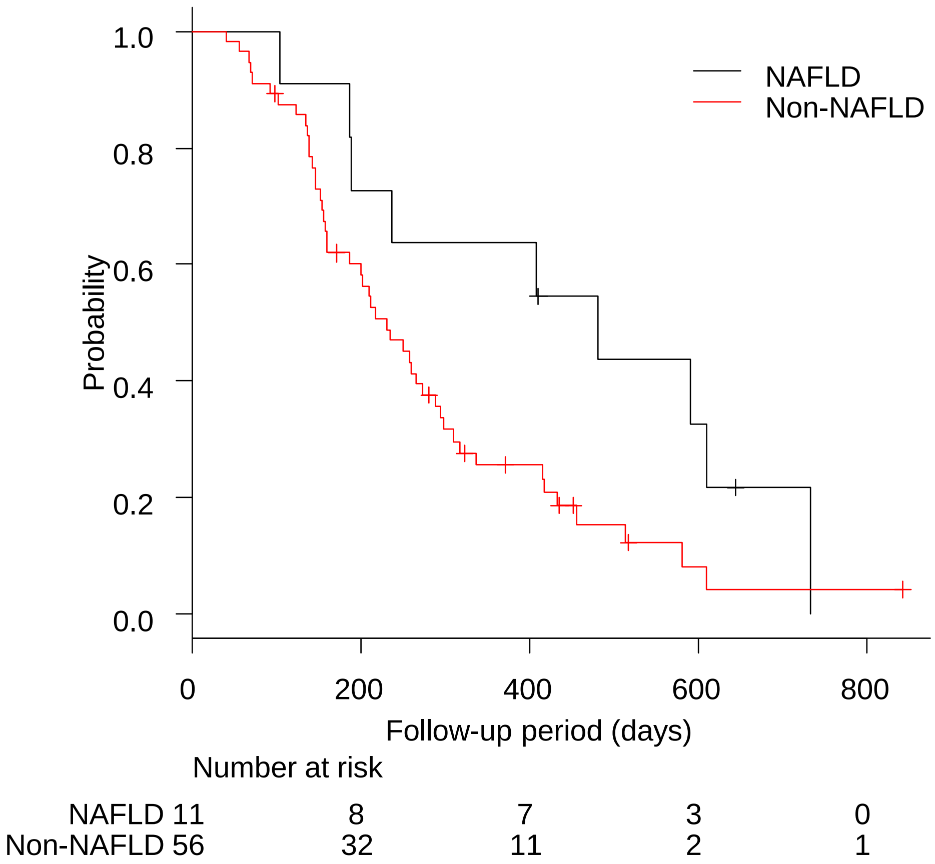

b)

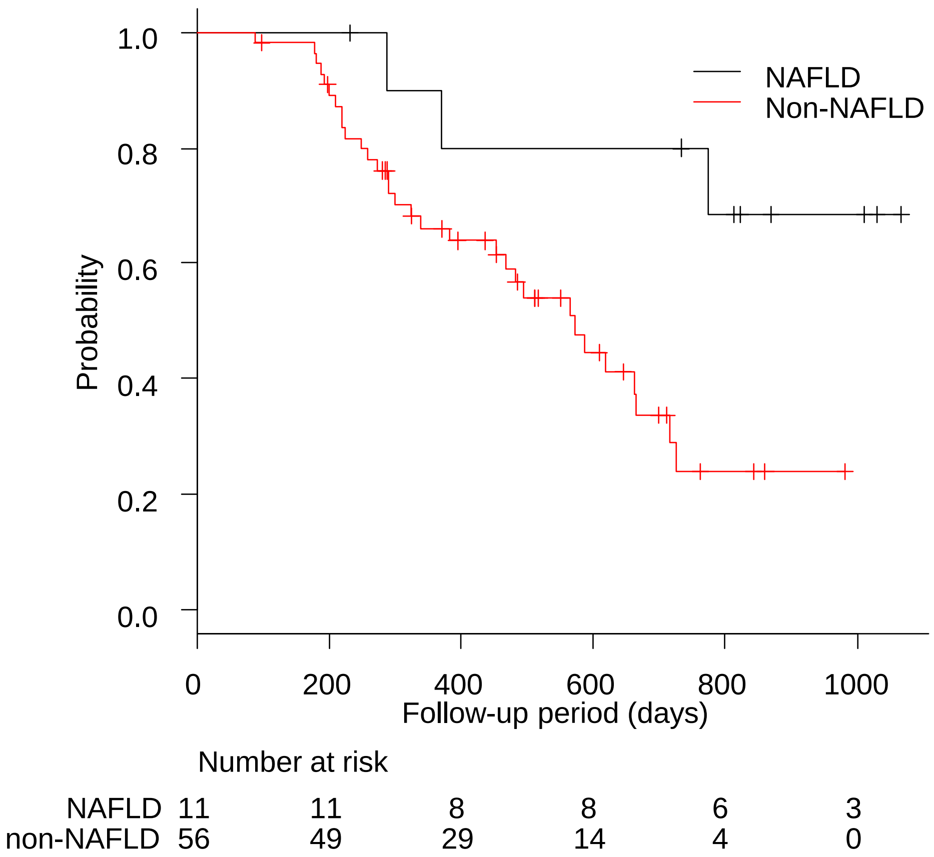

Supplement: Supplementary file 1 — Figure S1. Kaplan–Meier analysis of progression‐free survival among patients with advanced hepatocellular carcinoma treated with lenvatinib as the first‐line treatment according to etiology. (a) The PFS in the non‐NAFLD group was significantly longer than NAFLD group. Kaplan–Meier analysis of overall survival among patients with advanced hepatocellular carcinoma treated with lenvatinib as the first‐line treatment according to etiology. (b) The OS in the nonviral group was significantly longer than that in the viral group. NAFLD, nonalcoholic fatty liver disease; OS, overall survival. [file JGH3-5-1275-s002.pdf]
