# Supplementary figures and images for: Therapeutic efficacy of lenvatinib in nonviral unresectable hepatocellular carcinoma
Source: JGH Open. 2021 Oct 22;5(11):1275–83. doi: 10.1002/jgh3.12663 (PMC8593789; doi:10.1002/jgh3.12663)

Supplementary.2

a)

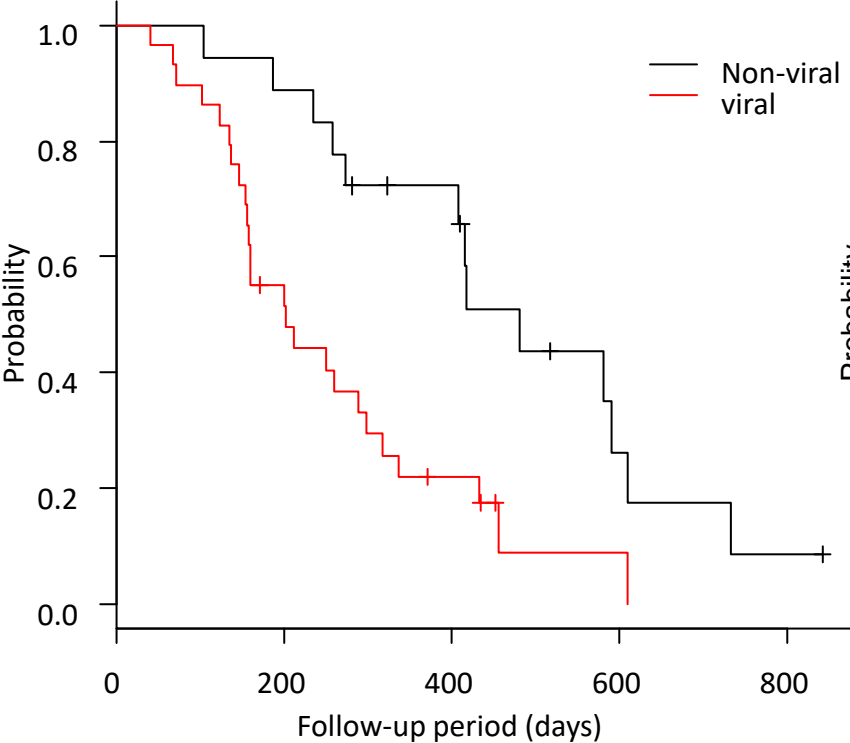

| Number at risk |    |    |    |   |   |
|----------------|----|----|----|---|---|
| Non-viral      | 18 | 16 | 11 | 3 | 1 |
| viral          | 29 | 15 | 5  | 1 | 0 |

b)

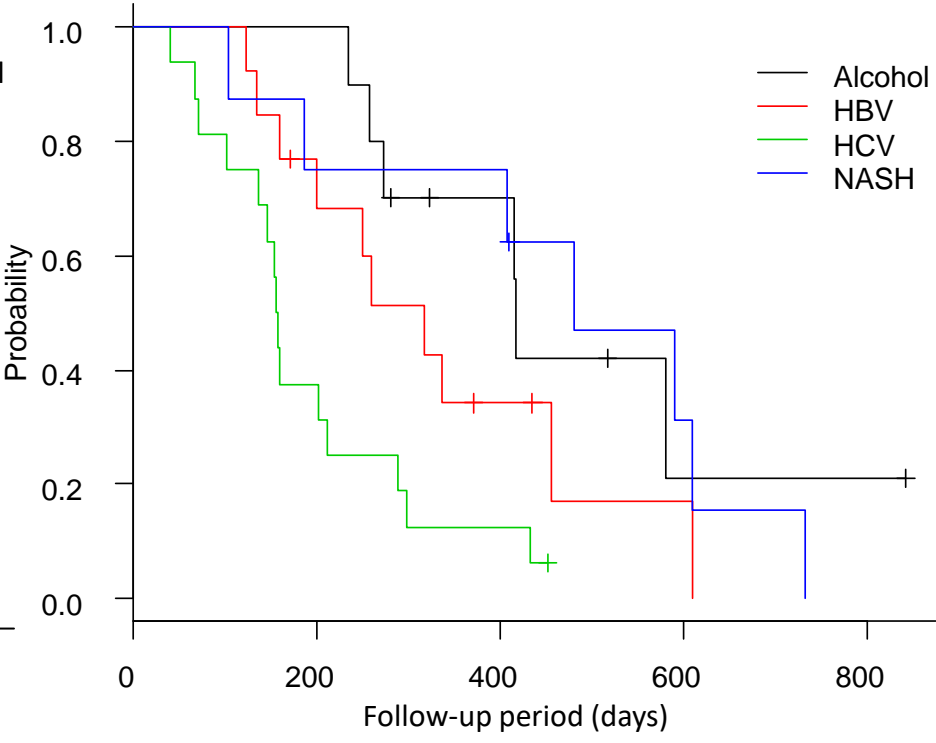

| Number at risk |    |    |   |   |   |
|----------------|----|----|---|---|---|
| Alcohol        | 10 | 10 | 5 | 1 | 1 |
| HBV            | 13 | 9  | 3 | 1 | 0 |
| HCV            | 16 | 6  | 2 | 0 | 0 |
| NASH           | 8  | 6  | 6 | 2 | 0 |

Supplement: Supplementary file 2 — Figure S2. Kaplan–Meier analysis of progression‐free survival among patients with advanced hepatocellular carcinoma treated with lenvatinib as the first‐line treatment according to etiology. (a) The PFS in the nonviral group was significantly longer than that in the viral group. (b) The PFS among alcohol, NASH, HBV, and HCV groups. NASH, nonalcoholic steatohepatitis; HBV, hepatitis B virus; HCV, hepatitis C virus; PFS, progression‐free survival. [file JGH3-5-1275-s001.pdf]

Supplementary.3

a)

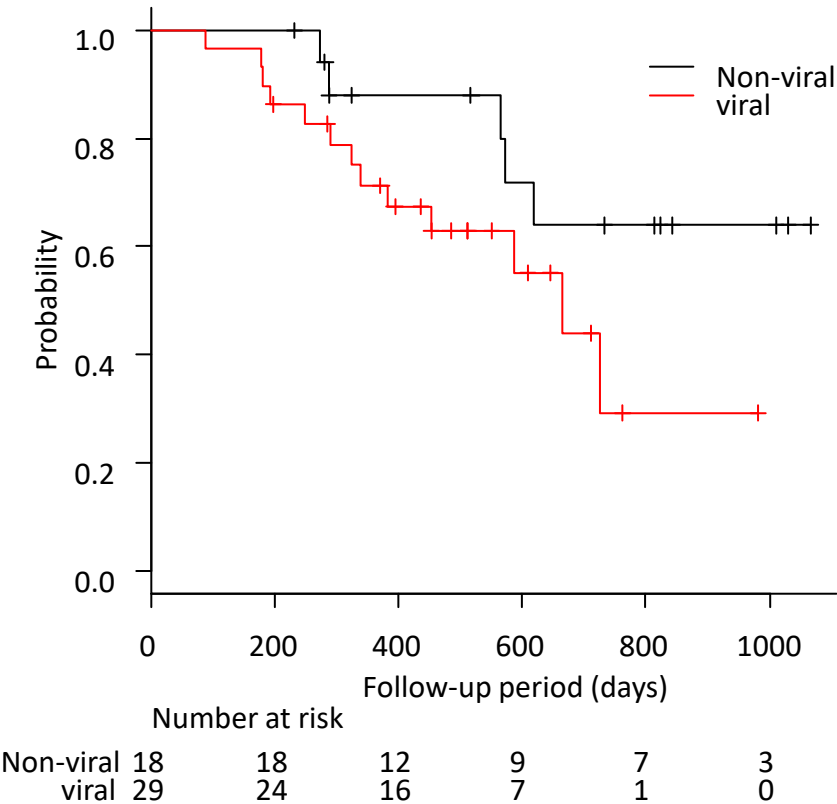

b)

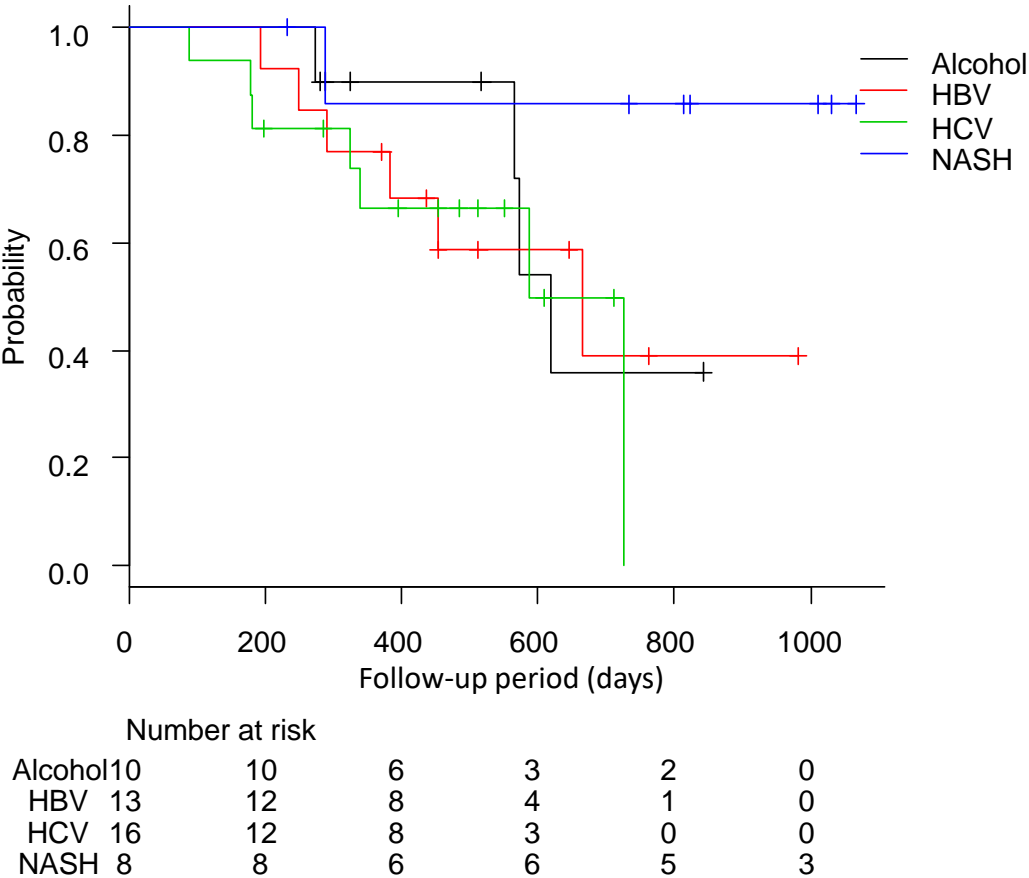

Supplement: Supplementary file 3 — Figure S3. Kaplan–Meier analysis of overall survival among patients with advanced hepatocellular carcinoma treated with lenvatinib as the first‐line treatment according to etiology. (a) The OS in the nonviral group was significantly longer than that in the viral group. (b) The OS among alcohol, NASH, HBV, and HCV groups. HBV, hepatitis B virus; HCV, hepatitis C virus; OS, overall survival. [file JGH3-5-1275-s004.pdf]
