# Supplementary material for: Therapeutic efficacy of lenvatinib in nonviral unresectable hepatocellular carcinoma
Source: JGH Open. 2021 Oct 22;5(11):1275–83. doi: 10.1002/jgh3.12663 (PMC8593789; doi:10.1002/jgh3.12663)
Supplement: Supplementary file 4 — Table S1. Characteristics of patients with unresectable advanced hepatocellular carcinoma treated with lenvatinib. Table S2. Univariate and multivariate analyses of the factors influencing progression‐free survival in first line. Table S3. Univariate and multivariate analyses of the factors influencing overall survival in first line. Table S4. Adverse events associated with lenvatinib treatment. [file JGH3-5-1275-s003.docx]

**Supplementary Table 1.** Characteristics of patients with unresectable advanced hepatocellular carcinoma treated with lenvatinib.

| **Characteristics** | **All**  **(*n* = 67)** | **Viral**  **(*n* = 45)** | **Non-viral**  **(*n* =22)** | ***p*-value** |
| --- | --- | --- | --- | --- |
| Age, median  [Quaritiles], (years) | 71  [66–77] | 70  [65–77] | 72  [70–77] | 0.09 |
| Sex (male/female), *n* | 51/16 | 34/11 | 17/5 | 1 |
| ECOG PS (0/1), *n* | 60/7 | 40/5 | 20/2 | 1 |
| Platelets, median  [Quaritiles], (10^4^/μl) | 14.5  [8.6–19.0] | 14.4  [10.2–18.6] | 16.9  [10.8–38.2] | 0.37 |
| M2BpGi  [Quaritiles] (C.O.I) | 1.44  [0.95–2.50] | 1.59  [0.89–3.02] | 1.2  [0.97–2.23] | 0.37 |
| Child-Pugh score (5/6/7/8), *n* | 38/29/0/0 | 27/18/0/0 | 11/11/0/0 | 0.6 |
| mALBI Grade (1/2a/2b/3), *n* | 26/20/21/0 | 20/11/14/0 | 6/9/7/0 | 0.31 |
| Number of intrahepatic lesions (None/1/2–7/> 7) | 0/12/25/30 | 0/6/15/24 | 0/6/10/6 | 0.11 |
| Maximum size of intrahepatic lesion (None/≤ 50/> 50) (mm) | 0/49/18 | 0/33/12 | 0/16/6 | 1 |
| Portal vein invasion (absent/present), *n* | 54/13 | 38/7 | 17/5 | 0.51 |
| Extrahepatic spread (absent/present), *n* | 52/15 | 34/11 | 18/4 | 0.76 |
| AFP, median  [Quaritiles] (ng/ml) | 24  [6–506] | 37  [6–1800] | 16  [6–334] | 0.22 |
| BCLC stage (B/C), *n* | 39/28 | 27/18 | 12/10 | 0.79 |
| Treatment line  (1^st^ line/2^nd^ line/3^rd^ line), n | 47/10/10 | 29/8/8 | 18/2/2 | 0.44 |
| Previous treatment times of TAE/TACE [Quaritiles] | 1  [1–2] | 1  [1–3] | 1  [1–2] | 0.26 |
| Initial dose of Lenvatinib (12/8/4), (mg), *n* | 36/30/1 | 21/23/1 | 15/7/0 | 0.22 |

*HBV*: hepatitis B virus, *HCV*: hepatitis C virus, *NBNC*: non B non C, *ECOG PS*: Eastern Cooperative Oncology Group performance status, *M2BPGi* mac-2 binding protein glycosylation isomer, *ALBI*: albumin-bilirubin, *AFP*: alpha-fetoprotein, *BCLC*: Barcelona Clinic Liver Cancer, *TAE/TACE*: transcatheter embolization/chemoembolization.

**Supplementary Table 2.** Univariate and multivariate analyses of the factors influencing progression-free survival in first line.

| **Variables** | **Category** | **No. of patients** | **Median PFS**  **(days)** | **Univariate** | **Multivariate** | |
| --- | --- | --- | --- | --- | --- | --- |
|  |  |  |  | ***P* value** | **Hazard ratio**  **(95% confidence interval)** | ***P* value** |
| Age, (years) | ≥70  <70 | 31  16 | 290  273 | 0.34 |  |  |
| Sex | Male  Female | 37  10 | 299  188 | 0.94 |  |  |
| Etiology | Non-viral  Viral | 18  29 | 482  203 | 0.0027 | 0.268  (0.127-0.568) | 0.00059 |
| ECOG PS | 1  0 | 4  43 | 420  290 | 0.55 |  |  |
| mALBI Grade | 1,2a  2b | 37  10 | 299  188 | 0.22 |  |  |
| Number of tumors | >7  ≤7 | 29  18 | 290  259 | 0.82 |  |  |
| Maximum size of tumor (mm) | ≥50  <50 | 14  33 | 188  317 | 0.77 |  |  |
| Portal vein invasion | Yes  No | 8  39 | 162  317 | 0.011 | 4.837  (1.905–12.28) | 0.00092 |
| Extrahepatic spread | Yes  No | 9  38 | 261  290 | 0.28 |  |  |
| AFP level (ng/mL) | ≥400  <400 | 12  35 | 213  299 | 0.82 |  |  |

ECOG PS, Eastern Cooperative Oncology Group performance status; AFP, alpha-fetoprotein; OS, overall survival; NA, not applicable

**Supplementary Table 3.**  Univariate and multivariate analyses of the factors influencing overall survival in first line.

| **Variables** | **Category** | **No. of patients** | **Median OS**  **(days)** | **Univariate** | **Multivariate** | |
| --- | --- | --- | --- | --- | --- | --- |
|  |  |  |  | ***P* value** | **Hazard ratio**  **(95% confidence interval)** | ***P* value** |
| Age, (years) | ≥70  <70 | 31  16 | NA  665 | 0.45 |  |  |
| Sex | Male  Female | 37  10 | 727  NA | 0.52 |  |  |
| Etiology | Non-viral  Viral | 18  29 | NA  665 | 0.04 | 0.344  (0.344-0.999) | 0.048 |
| ECOG PS | 1  0 | 4  43 | NA  665 | 0.22 |  |  |
| mALBI Grade | 1,2a  2b | 37  10 | 727  572 | 0.18 |  |  |
| Number of tumors | >7  ≤7 | 29  18 | 727  NA | 0.71 |  |  |
| Maximum size of tumor (mm) | ≥50  <50 | 14  33 | 665  727 | 0.72 |  |  |
| Portal vein invasion | Yes  No | 8  39 | 288  NA | 0.0011 | 6.816  (2.145–21.66) | 0.0011 |
| Extrahepatic spread | Yes  No | 9  38 | 587  NA | 0.33 |  |  |
| AFP level (ng/mL) | ≥400  <400 | 12  35 | 665  727 | 0.87 |  |  |

ECOG PS, Eastern Cooperative Oncology Group performance status; AFP, alpha-fetoprotein; OS, overall survival; NA, not applicable

**Supplementary Table 4.** Adverse events associated with Lenvatinib treatment.

| Event | All  (*n* = 67) | | Viral  *(n* = 45) | | Non-viral  (*n* = 22) | | *p*-value | |
| --- | --- | --- | --- | --- | --- | --- | --- | --- |
|  | **Any Grade** | **Grade3** | **Any Grade** | **Grade3** | **Any Grade** | **Grade3** | **Any Grade** | **Grade3** |
| Hypertension | 32  (47.8) | 8  (11.9) | 18  (40.0) | 4  (8.9) | 14  (63.6) | 4  (18.2) | 0.12 | 0.42 |
| Fatigue | 32  (47.8) | 4  (6.0) | 20  (44.4) | 3  (6.7) | 12  (54.5) | 1  (4.5) | 0.60 | 1 |
| Decreased appetite | 22  (32.8) | 1  (1.4) | 14  (31.1) | 1  (2.2) | 8  (36.3) | 0  (0) | 0.78 | 1 |
| Decreased platelet count | 14  (20.9) | 2  (3) | 9  (20.0) | 0  (0) | 5  (22.7) | 2  (9.1) | 1 | 0.10 |
| Palmar-plantar erythrodysesthesia | 19  (28.4) | 0  (0) | 14  (31.1) | 0  (0) | 5  (22.7) | 0  (0) | 0.57 | - |
| Proteinuria | 31  (46.3) | 13  (19.4) | 17  (37.8) | 8  (17.8) | 14  (63.6) | 5  (22.7) | 0.07 | 0.75 |
| Diarrhea | 10  (14.9) | 2  (3) | 9  (20.0) | 2  (4.4) | 1  (4.5) | 0  (0) | 0.15 | 1 |
| Increased blood bilirubin | 6  (9.0) | 0  (0) | 2  (4.4) | 0  (0) | 4  (18.1) | 0  (0) | 0.09 | - |
| Dysphonia | 10  (14.9) | 0  (0) | 5  (11.1) | 0  (0) | 5  (22.7) | 0  (0) | 0.68 | - |
| Elevated-aspartate aminotransferase | 7  (10.4) | 0  (0) | 6  (13.3) | 0  (0) | 1  (4.5) | 0  (0) | 0.17 | - |
| Hypothyroidism | 9  (13.4) | 0  (0) | 8  (17.8) | 0  (0) | 1  (4.5) | 0  (0) | 0.25 | - |
| Elevated-alanine  aminotransferase | 4  (6.0) | 0  (0) | 4  (8.9) | 0  (0) | 0  (0) | 0  (0) | 0.29 | - |

The *p*-values were calculated using Fisher’s exact test.
